# The 1:1 co-crystal of 2-bromo­naphthalene-1,4-dione and 1,8-di­hydroxy­anthracene-9,10-dione: crystal structure and Hirshfeld surface analysis

**DOI:** 10.1107/S2056989017005667

**Published:** 2017-04-21

**Authors:** Marlon D. L. Tonin, Simon J. Garden, Mukesh M. Jotani, Solange M. S. V. Wardell, James L. Wardell, Edward R. T. Tiekink

**Affiliations:** a Instituto de Química, Universidade Federal do Rio de Janeiro, Centro Tecnológica, Bloco A, Cidade Universitária, Ilha do Fundão, 21949-909 Rio de Janeiro, RJ, Brazil; b Department of Physics, Bhavan’s Sheth R. A. College of Science, Ahmedabad, Gujarat 380 001, India; c CHEMSOL, 1 Harcourt Road, Aberdeen AB15 5NY, Scotland; d Fundaçaö Oswaldo Cruz, Instituto de Tecnologia em Fármacos-Far Manguinhos, 21041-250 Rio de Janeiro, RJ, Brazil; e Department of Chemistry, University of Aberdeen, Old Aberdeen, AB24 3UE, Scotland; f Research Centre for Chemical Crystallography, School of Science and Technology, Sunway University, 47500 Bandar Sunway, Selangor Darul Ehsan, Malaysia

**Keywords:** crystal structure, co-crystal, naphthalene-1,4-dione, di­hydroxy­anthracene-9,10-dione, Hirshfeld surface analysis

## Abstract

The asymmetric unit of the title co-crystal, C_10_H_5_BrO_2_·C_14_H_8_O_4_ [systematic name: 2-bromo-1,4-di­hydro­naphthalene-1,4-dione–1,8-dihy­droxy-9,10-di­hydro­anthracene-9,10-dione (1/1)], features one mol­ecule of each coformer. The 2-bromo­naphtho­quinone mol­ecule is almost planar [r.m.s deviation of the 13 non-H atoms = 0.060 Å, with the maximum deviations of 0.093 (1) and 0.099 (1) Å being for the Br atom and a carbonyl-O atom, respectively]. The 1,8-di­hydroxy­anthra­quinone mol­ecule is planar (r.m.s. deviation for the 18 non-H atoms is 0.022 Å) and features two intra­molecular hy­droxy-O—H⋯O(carbon­yl) hydrogen bonds. Dimeric aggregates of 1,8-di­hydroxy­anthra­quinone mol­ecules assemble through weak inter­molecular hy­droxy-O—H⋯O(carbon­yl) hydrogen bonds. The mol­ecular packing comprises stacks of mol­ecules of 2-bromo­naphtho­quinone and dimeric assembles of 1,8-di­hydroxy­anthra­quinone with the shortest π–π contact within a stack of 3.5760 (9) Å occurring between the different rings of 2-bromo­naphtho­quinone mol­ecules. The analysis of the Hirshfeld surface reveals the importance of the inter­actions just indicated but, also the contribution of additional C—H⋯O contacts as well as C=O⋯π inter­actions to the mol­ecular packing.

## Abstract 2

The 1:1 co-crystal comprising two fused-ring mol­ecules features significant hydrogen bonding between the 1,8-di­hydroxy­anthra­quinone coformers with the main links between the resulting dimeric aggregates and the bromo­naphtho­quinone coformer being of the type C—H⋯O.

## Chemical context   

The formation of co-crystals is one of the major activities of crystal engineering endeavours and is motivated by various considerations. The concept of non-covalent derivatization of active pharmaceutical ingredients (API’s) by this technology, in the hope of producing new formulations with improved bio-availability, *etc*. is a prominent motivation for investigation (Duggirala *et al.*, 2016 ▸; Bolla & Nangia, 2016 ▸). Over and above this are applications ranging from enhancing non-linear optical materials, crystallization of materials that normally do not crystallize, optical resolution, *etc*. (Aakeröy, 2015 ▸). The above notwithstanding, the title co-crystal, (I), was isolated serendipiously during attempts to react 2-bromo­naphtho­quinone with 1,8-di­hydroxy­anthra­quinone. Subsequently, it was shown that an equimolar ethyl acetate (or ethanol) solution of 2-bromo­naphtho­quinone and 1,8-di­hydroxy­anthra­quinone could be co-crystallized to give the same product. Herein, the crystal and mol­ecular structures of (I) are described along with a detailed analysis of the supra­molecular association by means of an analysis of the Hirshfeld surfaces.
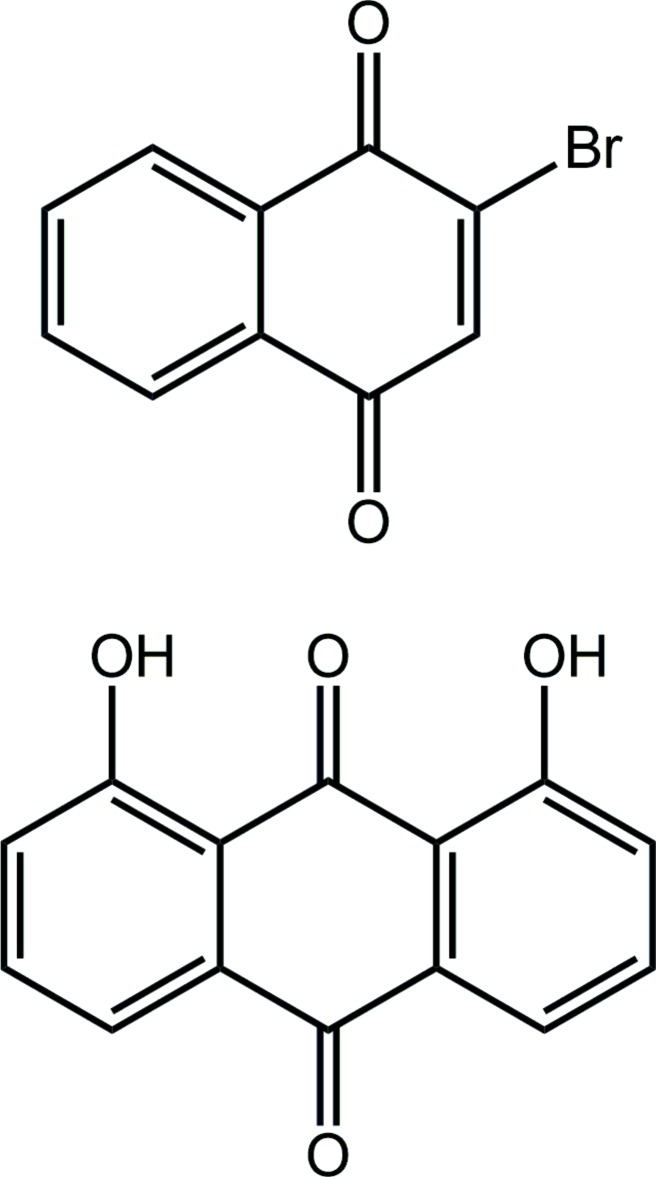


## Structural commentary   

**Figure 1:**
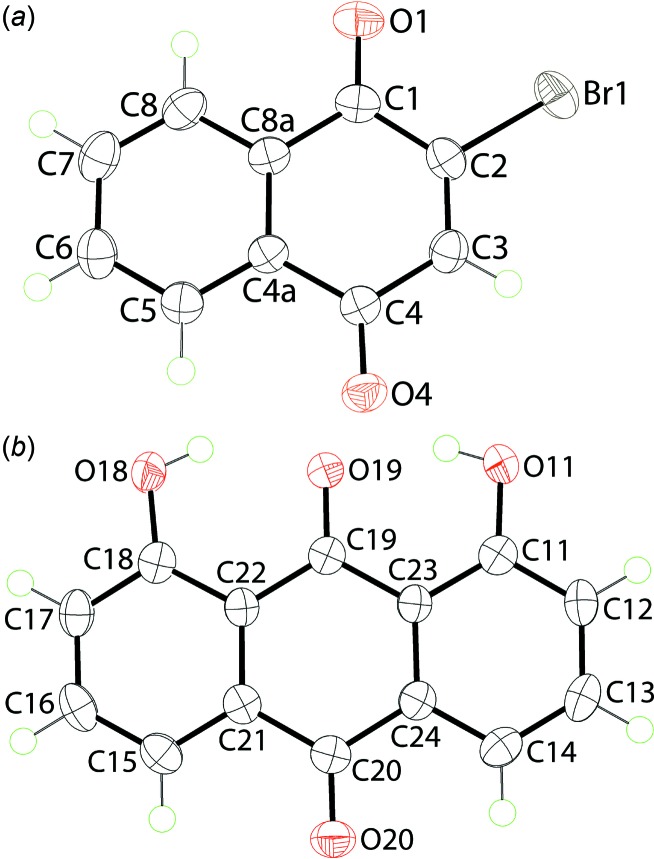
The mol­ecular structures of (*a*) 2-bromo­naphtho­quinone and (*b*) 1,8-di­hydroxy­anthra­quinone, *i.e*. the coformers comprising the asymmetric unit of (I), showing the atom-labelling scheme and displacement ellipsoids at the 70% probability level.

The mol­ecular structures of the constituents of (I) are shown in Fig. 1 ▸, the asymmetric unit comprising one mol­ecule each of 2-bromo­naphtho­quinone, Fig. 1 ▸ *a*, and 1,8-di­hydroxy­anthra­quinone, Fig. 1 ▸ *b*. The six carbon atoms comprising the cyclo­hexa-2,5-diene-1,4-dione ring of the naphtho­quinone mol­ecule are not strictly planar with the r.m.s. deviation being 0.030 Å; the maximum deviations are 0.025 (1) and −0.031 (2) Å for the C4a and C4 atoms, respectively. The appended Br1, O1 and O4 atoms lie, respectively, 0.077 (1), 0.078 (1) and −0.117 (1) Å out of the plane with the Br1 atom lying to one side of the ring and the carbonyl-O atoms to the other. Overall, the r.m.s. deviation for the best plane defined by the 13 non-H atoms comprising the naphtho­quinone mol­ecule is 0.060 Å, with the maximum deviations being 0.093 (1) Å for atom Br1 and −0.099 (1) Å for the O4 atom, again with these atoms lying to opposite sides of the plane. With respect to the anthra­quinone mol­ecule, the r.m.s. deviation for the 18 non-H atoms is 0.022 Å with the maximum deviations being 0.039 (2) Å for C(13) and 0.026 (1) Å for the C19 and C23 atoms. As seen from Fig. 1 ▸ *b*, the hy­droxy-H atoms are orientated to be proximate to the centrally located carbonyl-O atom to form intra­molecular hy­droxy-O—H⋯O(carbon­yl) hydrogen-bonds, Table 1 ▸.

## Supra­molecular features   

**Figure 2:**
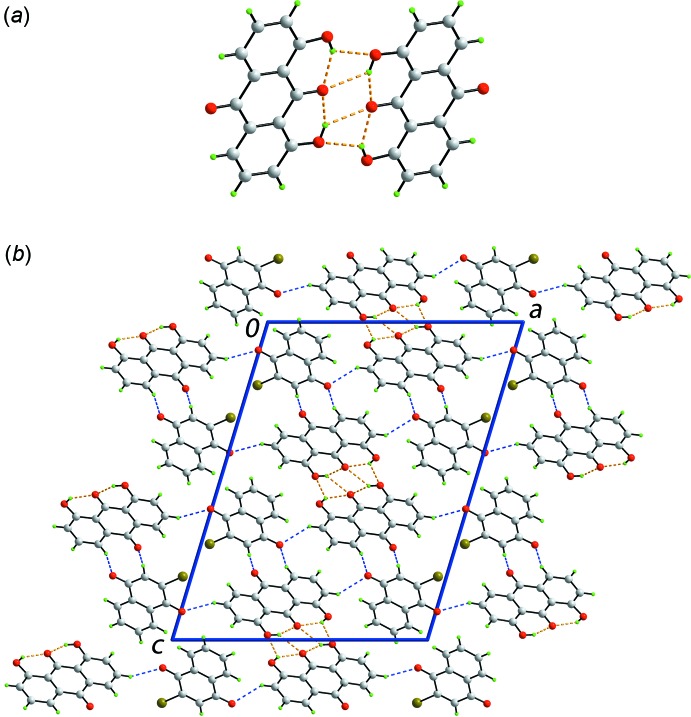
The mol­ecular packing in (I): (*a*) dimeric aggregate comprising centrosymmetrically related 1,8-di­hydroxy­anthra­quinone mol­ecules connected by hy­droxy-O—H⋯O(carbon­yl) hydrogen bonds and (*b*) a view of the unit-cell contents in projection down the *b* axis. The O—H⋯O and phenyl-C—H⋯O(carbon­yl) inter­actions are shown as orange and blue dashed lines, respectively.

In addition to the intra­molecular hy­droxy-O—H⋯O(carbon­yl) hydrogen-bonds in the anthra­quinone mol­ecule, both hy­droxy-H atoms from weaker inter­molecular hydrogen-bonds with a centrosymmetrically related mol­ecule indicating each hy­droxy-H atom is bifurcated, Table 1 ▸. The resulting dimeric aggregate, Fig. 2 ▸ *a*, is connected by a centrosymmetric planar, eight-membered {⋯HO⋯O⋯H}_2_ synthon which incorporates two transannular hy­droxy-O—H⋯O(carbon­yl) hydrogen bonds. The dimeric aggregates stack along the *b* axis being surrounded by two columns of similar dimeric aggregates and six columns comprising naphtho­quinone mol­ecules, Fig. 2 ▸ *b*. Connections between columns, leading to a three-dimensional architecture, are of the type *sp* ^2^-C—H⋯O(carbon­yl) and involve all the remaining carbonyl-O atoms with the O atom of the naphtho­quinone-C4=O4 moiety forming two such contacts, Table 1 ▸. Within columns comprising mol­ecules of naphtho­quinone, π–π stacking inter­actions are noted, *i.e*. between the (C1–C4,C4a,C8a) and (C4a,C5–C8,C8a) rings with the inter-centroid separation being 3.5760 (9) Å and the angle of inclination being 1.64 (7)° for symmetry operation *x*, −1 + *y*, *z*. The closest comparable inter­action within the stack of anthra­quinone mol­ecules is 4.1013 (9) Å, *i.e*. between (C15–C21) and (C19–C24) rings; angle of inclination = 0.65 (7)° for symmetry operation: *x*, −1 + *y*, *z*.

## Hirshfeld surface analysis   

**Figure 3:**
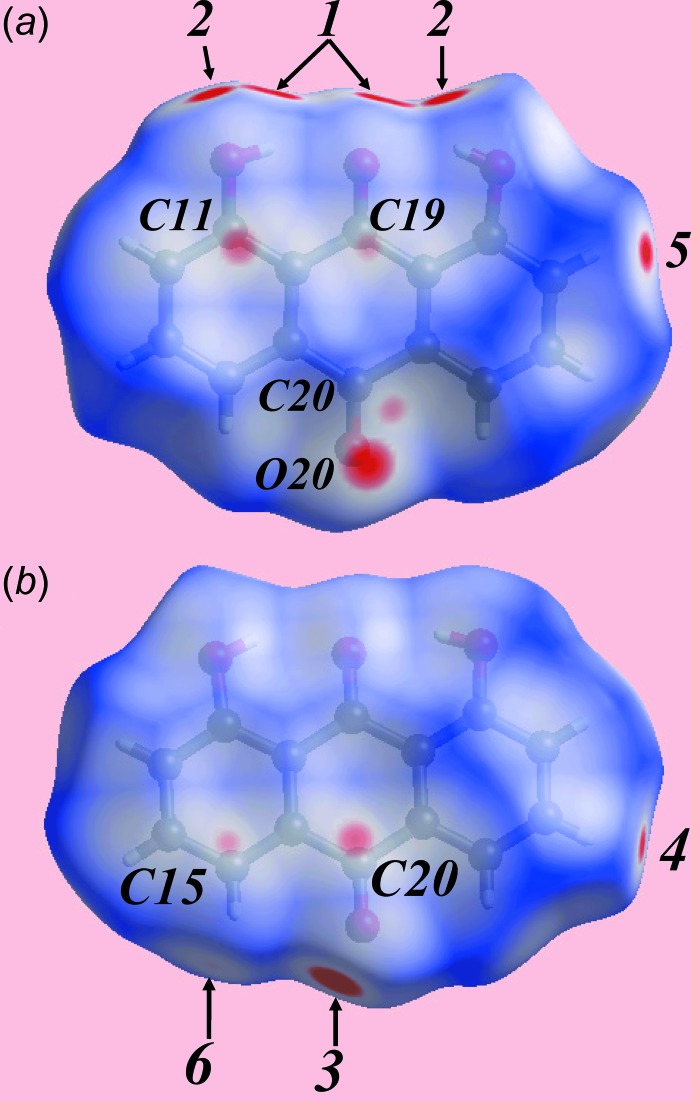
Two views of the Hirshfeld surface for the anthra­quinone mol­ecule in (I) mapped over *d*_norm_ over the range −0.120 to 1.190 au.

**Figure 5:**
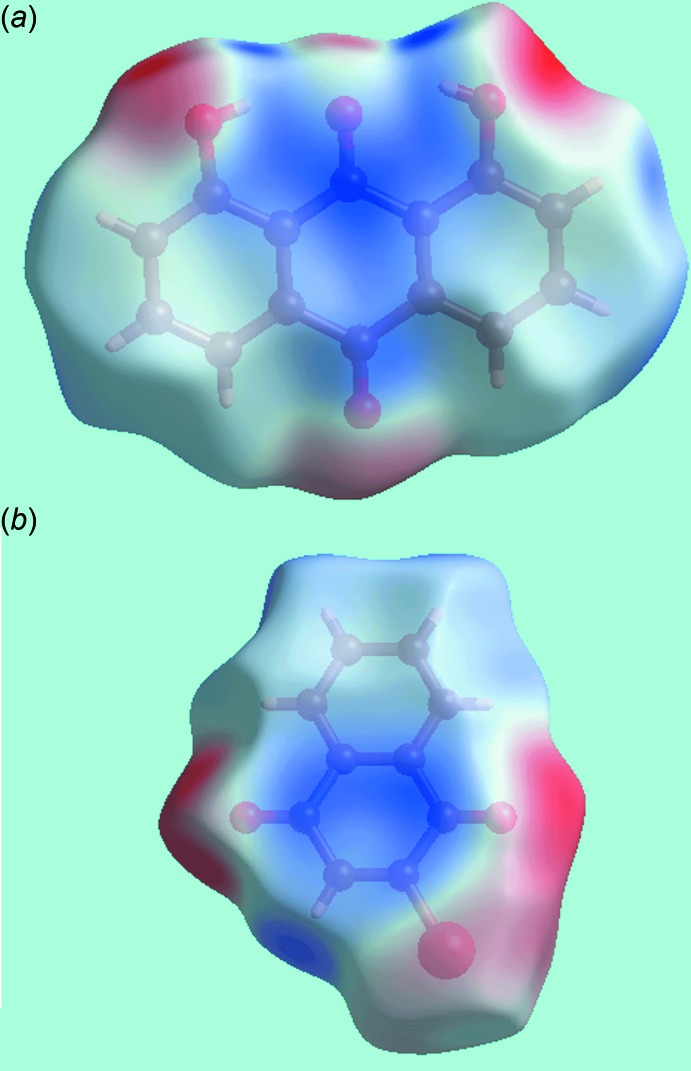
Views of the Hirshfeld surfaces for the (*a*) anthra­quinone and (*b*) naphtho­quinone mol­ecules in (I) mapped over the electrostatic potential in the range ±0.059 au. The red and blue regions represent negative and positive electrostatic potentials, respectively.

The Hirshfeld surface analysis of title 1:1 co-crystal, (I), was performed as per recent publications on co-crystals (Syed, Jotani, Halim *et al.*, 2016 ▸; Syed, Halim, Jotani *et al.*, 2016 ▸) and provides more detailed information on the supra­molecular association formed by the individual coformers and overall packing in the crystal. The Hishfeld surfaces are mapped over *d*_norm_, Figs. 3 ▸ and 4 ▸, the calculated electrostatic potential, Figs. 5 ▸ and 6 ▸, and shape-index, Figs. 7 ▸ and 8 ▸.

**Figure 6:**
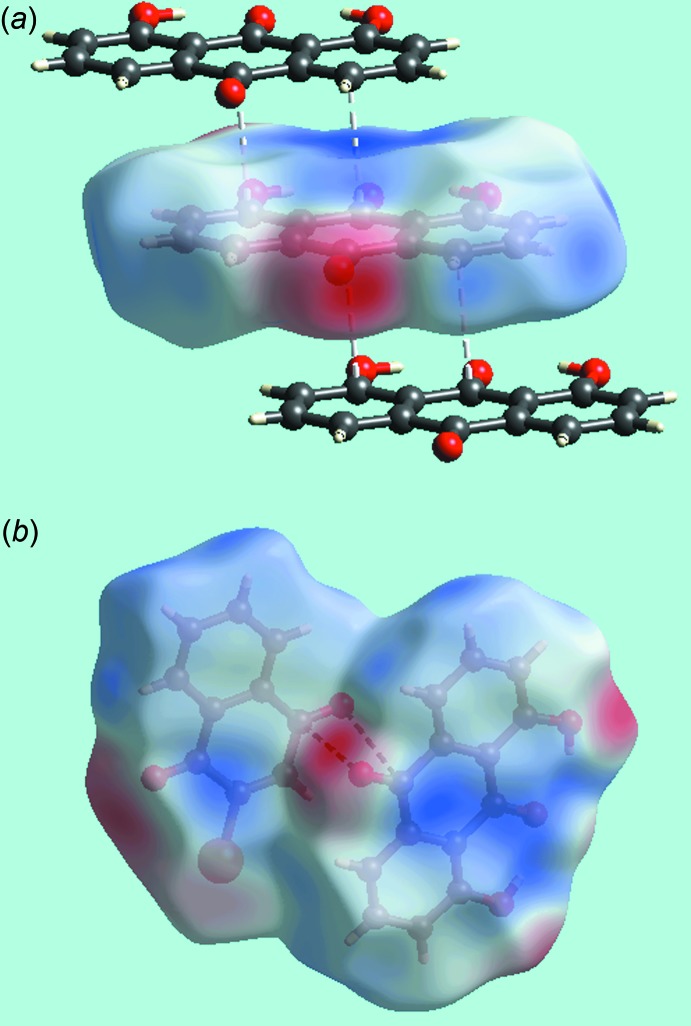
Views of Hirshfeld surfaces for the mol­ecules in (I) mapped over the electrostatic potential highlighting (*a*) short inter­atomic C⋯C contacts as with white dashed lines in the stacking of anthra­quinone mol­ecules in the range ±0.059 au and (*b*) short inter­atomic C⋯O/O⋯C contacts as black dashed lines between approximately co-planar anthra­quinone and naphtho­quinone mol­ecules in the range ±0.060 au.

**Figure 7:**
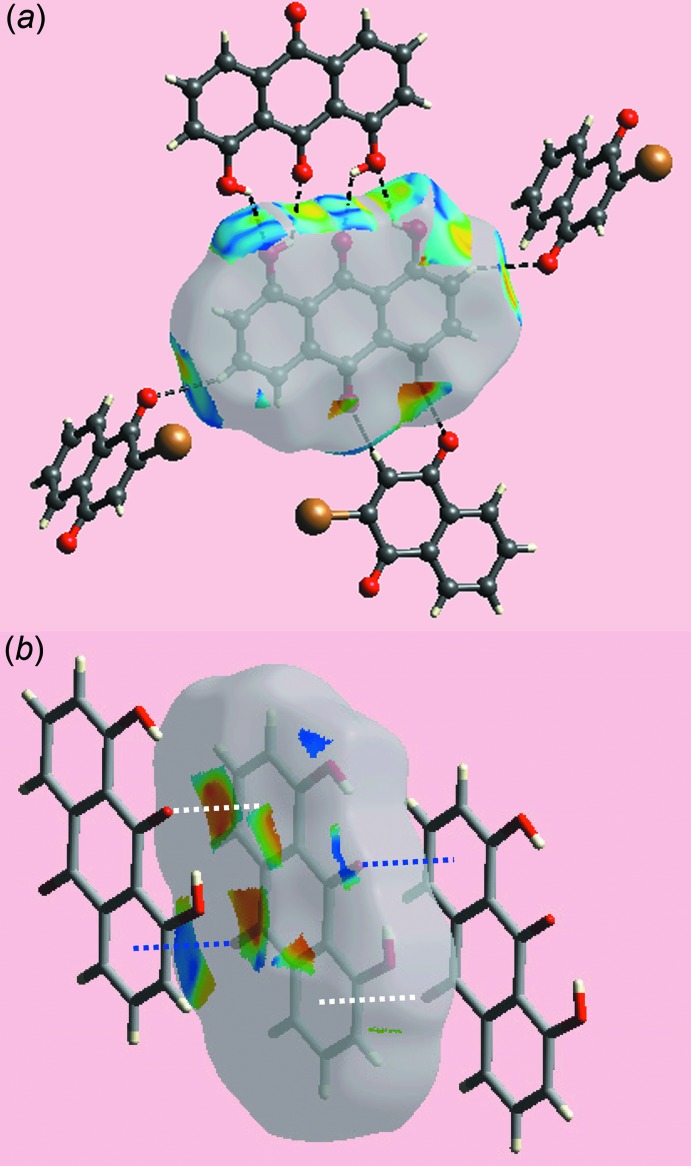
Views of Hirshfeld surface for a reference anthra­quinone mol­ecule in (I) mapped over the shape-index property highlighting: (*a*) O—H⋯O and C—H⋯O inter­actions as black dashed lines and (*b*) C—O⋯ π and reciprocal π⋯O—C inter­actions as blue and white dotted lines, respectively.

The donors and acceptors of inter­molecular hy­droxy-O—H⋯O(carbon­yl) hydrogen-bonds between anthra­quinone mol­ecules are viewed as bright-red spots labelled with ‘1’ and ‘2’ on the Hirshfeld surfaces mapped over *d*_norm_ in Fig. 3 ▸ *a*. On the Hirshfeld surface mapped over the calculated electrostatic potential, the respective donors and acceptors appear as the blue (positive potential) and red regions (negative potential) in Fig. 5 ▸ *a*. The presence of faint-red spots near carbon atoms C11, C19, Fig. 3 ▸ *a*, and near the atoms C15 and C20, Fig. 3 ▸ *b*, also indicate the links between mol­ecules through short inter-atomic C⋯C contacts, Table 2 ▸. These short contacts are also illustrated by white dashed lines in Fig. 6 ▸ *a*. Links between the coformers involving their carbonyl-C4=O4 and C20=O20 groups through short inter­atomic C⋯O/O⋯C contacts, Table 2 ▸, are viewed as a pair of bright- and faint-red spots near these atoms in Fig. 3 ▸ *b* and 4*b*. This is also illustrated by the black dashed lines on the Hirshfeld surface mapped over electrostatic potential in Fig. 6 ▸ *b*. The donors and acceptors of inter­molecular C—H⋯O(carbon­yl) inter­actions can be viewed as bright-red spots having labels ‘3’–‘5’ in Figs. 3 ▸ and 4 ▸, and as blue and red regions, respectively, in Fig. 5 ▸. The comparatively weak anthra­quinone-C15—H⋯O4 hydrogen bond is represented with faint-red spots near these atoms in Fig. 3 ▸ *b* and 4*a*, labelled with ‘6’. The immediate environments about reference anthra­quinone and naphtho­quinone mol­ecules within shape-index-mapped Hirshfeld surfaces highlighting inter­molecular O—H⋯O, C—H⋯O, π–π stacking and C—O⋯π inter­actions influential on the packing are illustrated in Figs. 7 ▸ and 8 ▸.

**Figure 9:**
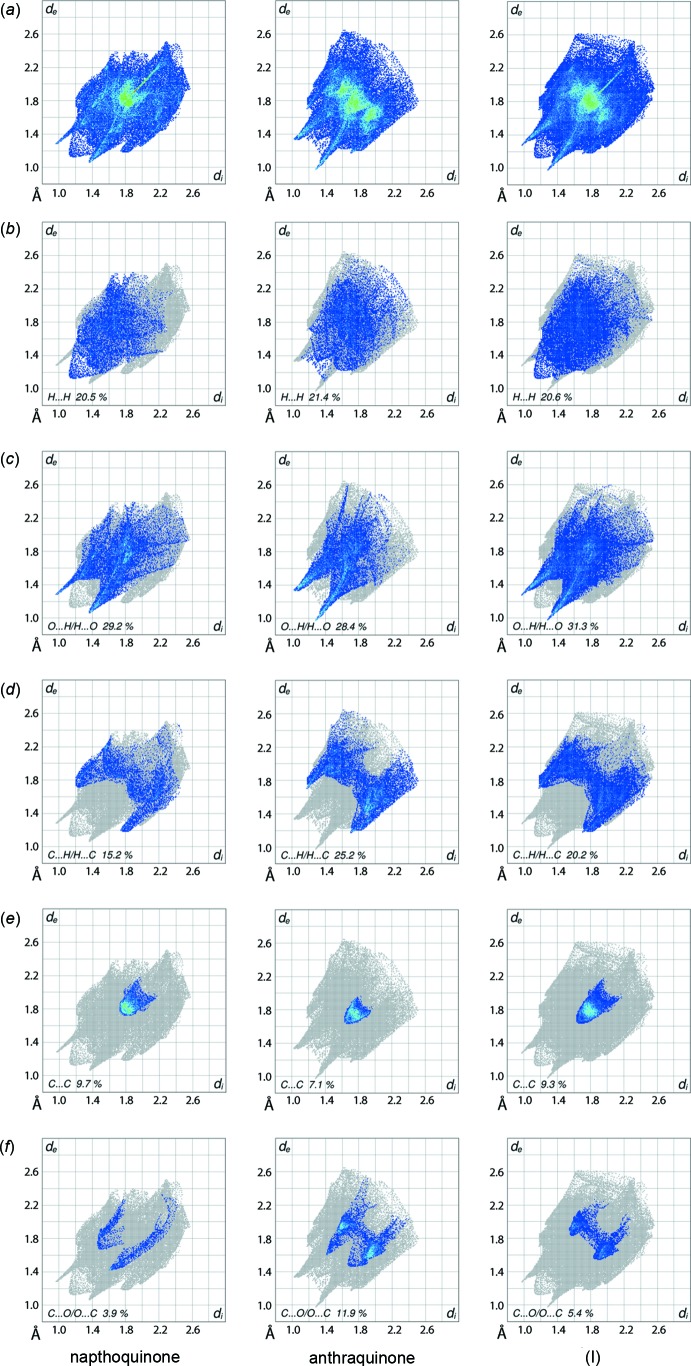
(*a*) The full two-dimensional fingerprint plots for the individual naphtho­quinone and anthra­quinone mol­ecules and the overall co-crystal (I), and fingerprint plots delineated into (*b*) H⋯H, (*c*) O⋯H/H⋯O, (*d*) C⋯H/H⋯C, (*e*) C⋯C and (*f*) C⋯O/O⋯C contacts.

The two-dimensional fingerprint plots for the individual naphtho­quinone and anthra­quinone mol­ecules, and for the overall co-crystal are illustrated in Fig. 9 ▸ *a*. The plots delineated into H⋯H, O⋯H/H⋯O, C⋯H/H⋯C, C⋯C and C⋯O/O⋯C contacts (McKinnon *et al.*, 2007 ▸) are shown in Fig. 9 ▸ *b*–*f*, respectively; the relative contributions from various contacts to the Hirshfeld surfaces are qu­anti­tatively summarized in Table 3 ▸. The different immediate environments of inter­molecular inter­actions around the naphtho­quinone and anthra­quinone coformers result in different shapes and a distinct distribution of points in the respective delineated fingerprint plots: there is a clear distinction between these and those for the overall co-crystal.

The fingerprint plots delineated into H⋯H contacts arise from relatively low percentage contributions to their respective Hirshfeld surfaces, Table 3 ▸, as a result of their relatively their low contents in the mol­ecules and the involvement of many hydrogen atoms in specific inter­molecular inter­actions. The presence of short inter­atomic H⋯H contacts between naphtho­quinone-H8 atoms, Table 2 ▸, is evident in the respective plot as a single peak at *d*_e_ + *d*_i_ ∼ 2.2 Å.

The donors and acceptors of the naphtho­quinone-H3 and anthra­quinone-O20(carbon­yl) atoms are viewed as a thin, long spike at *d*_e_ + *d*_i_ ∼ 2.2 Å in each of the fingerprint plots of O⋯H/H⋯O contacts, Fig. 9 ▸ *c*; the spikes for the donor and acceptor inter­actions are viewed separately in the plots for the naphtho­quinone and anthra­quinone coformers, respectively. The O—H⋯O inter­actions instrumental in linking anthra­quinone mol­ecules are evident in the respective O⋯H/H⋯O delineated plot, Fig. 9 ▸ *c*, and is characterized by a pair of short spikes at *d*_e_ + *d*_i_ ∼ 2.3 Å where in the acceptor spike is merged within the plot of the aforementioned C3—H⋯O^ii^ inter­action. The other inter­molecular C—H⋯O contacts involving anthra­quinone-H13 and -H17, and naphtho­quinone-O1 and -O4(carbon­yl) atoms are viewed as a pair of short spikes at *d*_e_ + *d*_i_ ∼ 2.4 Å in the donor and acceptor regions of their respective plots in Fig. 9 ▸ *c*. The points corresponding to anthra­quinone-C15—H15⋯O4(carbon­yl) inter­actions and other short inter­atomic O⋯H contacts, Table 2 ▸, are merged within the plots.

A pair of short peaks at *d*_e_ + *d*_i_ < 2.9 Å, *i.e*. less than sum of their van der Waals radii, in the fingerprint plot delineated into C⋯H/H⋯C contacts for anthra­quinone, Fig. 9 ▸ *d*, are indicative of short inter­atomic C⋯H contacts, Table 2 ▸, in the crystal. The remaining inter­atomic C⋯H/H⋯C contacts in the crystal are beyond van der Waals separations but still make notable contributions to the Hirshfeld surfaces. The 9.7% contribution from C⋯C contacts to the Hirshfeld surface of the naphtho­quinone coformer is the result of π–π stacking inter­action between its symmetry related (C1–C4,C4a,C8a) and (C4a,C5–C8,C8a) rings and is highlighted as the parabolic distribution of points in Fig. 9 ▸ *e*, having high density at around *d*_e_ = *d*_i_ ∼ 1.8 Å. The parabolic distribution of points with the peak at *d*_e_ = *d*_i_ ∼ 1.6 Å in the plot for the anthra­quinone coformer, Fig. 9 ▸ *e*, indicates links between these mol­ecules through short inter­atomic C⋯C contacts along the *b* axis. The presence of C⋯C contacts in (I) results in an overall 9.3% contribution to the Hirshfeld surface.

The 3.9% contribution from C⋯O/O⋯C contacts to the Hirshfeld surface for the naphtho­quinone mol­ecule, Fig. 9 ▸ *f*, results from short, inter-atomic C⋯O/O⋯C contacts whereas the 11.9% contribution from C⋯O/O⋯C contacts for the anthra­quinone mol­ecule has a contribution from C=O⋯π inter­actions involving carbony-O19 and -O20 atoms and (C11–C14,C24,C23) and (C15–C18, C22, C21) rings, Table 4 ▸. Most of these features disappear in the overall fingerprint plot delineated into these contacts with only features due to the C=O⋯π inter­actions remaining, Fig. 9 ▸ *f*.

Although the naphtho­quinone-bromide substituent makes a notable contribution to the Hirshfeld surface, Table 3 ▸, it does not form inter-atomic contacts with other atoms less than sum of the respective van der Waals radii. Therefore, it exerts no significant influence on the packing. The small contribution from O⋯O contacts also has a negligible effect on the packing.

## Database survey   

The coformers comprising (I) are relatively unexplored in the crystallographic literature (Groom *et al.*, 2016 ▸). For example, the structure of 2-bromo­naphtho­quinone has only been reported on one previous occasion, namely in its pure form (Gaultier & Hauw, 1965 ▸). This structure presents the same features as the mol­ecule in (I) with the r.m.s deviation of the 13 fitted atoms being 0.059 Å, *cf*. 0.060 Å in (I). More attention has been directed towards 1,8-di­hydroxy­anthra­quinone. The structure of the pure mol­ecule was originally reported in 1965 (Prakash, 1965 ▸) and a recent study focussed upon the several polymorphic forms of this compound (Rohl *et al.*, 2008 ▸). In all known forms of 1,8-di­hydroxy­anthra­quinone, an essentially planar mol­ecule is observed along with the two intra­molecular hy­droxy-O—H⋯O(carbon­yl) hydrogen-bonds persisting as in (I). A co-crystal of 1,8-di­hydroxy­anthra­quinone is also known, *i.e*. a 3:1 co-crystal with acetic acid (Cheuk *et al.*, 2015 ▸). This structure is particularly notable in that there are six independent 1,8-di­hydroxy­anthra­quinone mol­ecules in the asymmetric unit, each with the same conformation as in the parent compound and in (I), along with two independent acetic acid mol­ecules.

## Synthesis and crystallization   

Compound (I) was isolated during attempts to chemically bond 2-bromo­naphtho­quinone and 1,8-di­hydroxy­anthra­quinone under basic conditions. Upon work up of the reaction mixture, the crude material was obtained after evaporation of all the volatiles. This was filtered through a short column of silica gel eluting with CH_2_Cl_2_/hexane (1:1 *v*/*v*) and a single, yellow fraction was collected. After evaporation of the solvent under reduced pressure, a yellow solid was obtained. This was recrystallized from ethyl acetate solution to give small orange–red crystals with yields of 78–85% based upon the qu­antity of 1,8-di­hydroxy­anthra­quinone initially used. Notably, the substrates 2-bromo­naphtho­quinone and 1,8-di­hydroxy­anthra­quinone could not be chromatographically distinguished as they ran with equivalent *R*_f_’s in a wide range of solvents and solvent mixtures. NMR spectra (^1^H and ^13^C) were consistent with a one to one mixture of the same components as there was no deviation of chemical shifts in comparison to the spectra of the individual components. A sample of the co-crystal material had a well defined melting point of 413–414 K, which is inter­mediate between the melting points of the pure components 2-bromo­naphtho­quinone, 405–406 K (Brimble *et al.*, 2007 ▸) and 1,8-di­hydroxy­anthra­quinone, 465–466 K (Cameron *et al.*, 1982 ▸).

## Refinement   

Crystal data, data collection and structure refinement details are summarized in Table 5 ▸. Carbon-bound H atoms were placed in calculated positions (C—H = 0.95 Å) and were included in the refinement in the riding-model approximation, with *U*_iso_(H) set to 1.2*U*_eq_(C). The O-bound H atoms were located from a difference map but refined with O—H = 0.84±0.01 Å and *U*_iso_(H) = 1.5*U*_eq_(O).

## Supplementary Material

Crystal structure: contains datablock(s) I, global. DOI: 10.1107/S2056989017005667/wm5383sup1.cif

Structure factors: contains datablock(s) I. DOI: 10.1107/S2056989017005667/wm5383Isup2.hkl

Supporting information file. DOI: 10.1107/S2056989017005667/wm5383Isup3.cml

CCDC reference: 1543933

Additional supporting information:  crystallographic information; 3D view; checkCIF report

## Figures and Tables

**Figure 4:**
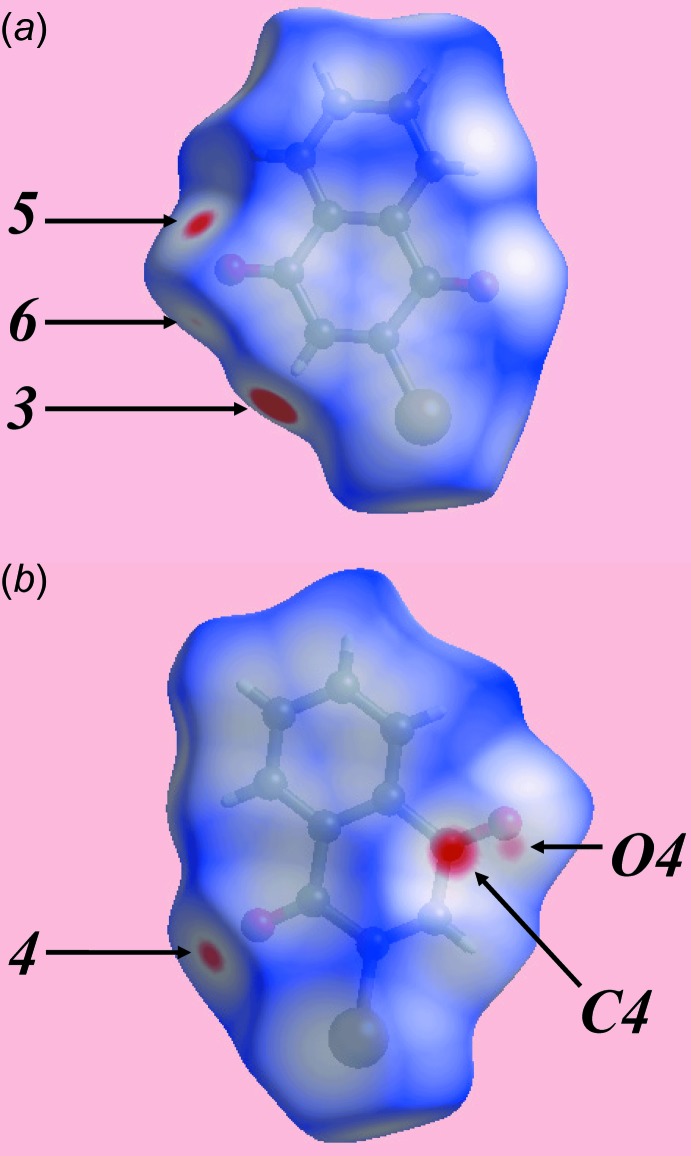
Two views of the Hirshfeld surface for the naphtho­quinone mol­ecule in (I) mapped over *d*_norm_ over the range −0.125 to 1.157 au.

**Figure 8:**
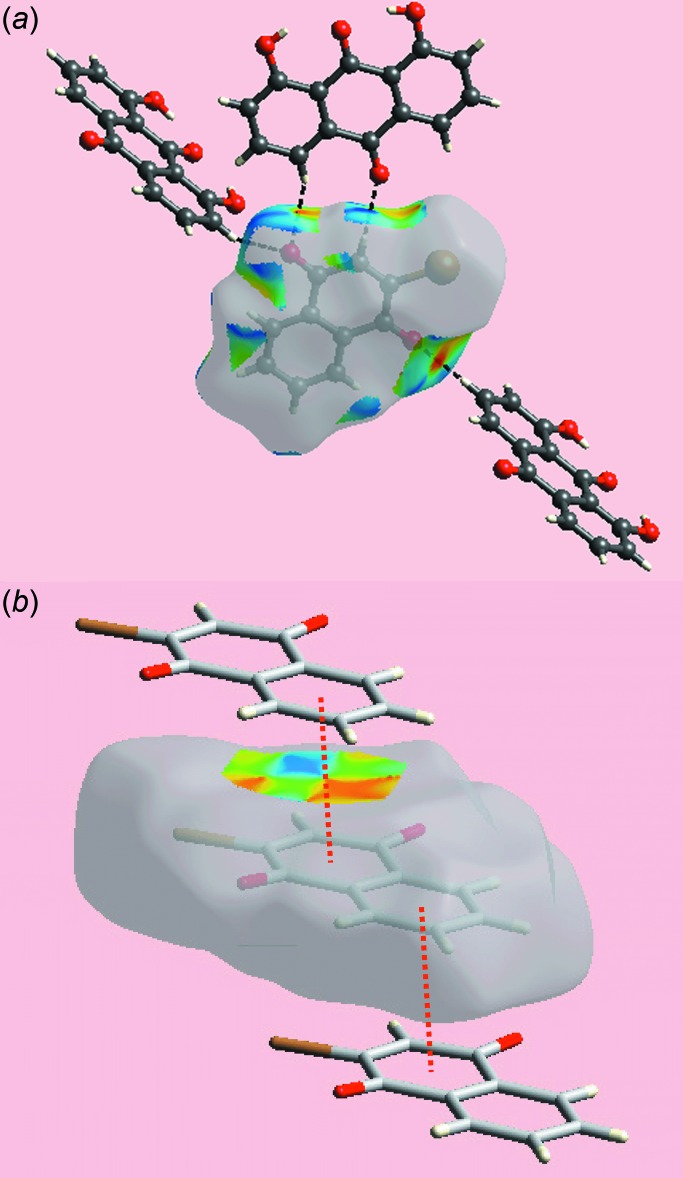
Views of Hirshfeld surface for a reference naphtho­quinone mol­ecule in (I) mapped over the shape-index property highlighting: (*a*) C—H⋯O inter­actions as black dashed lines and (*b*) π–π stacking inter­action as red dotted lines.

**Table 1:** Hydrogen-bond geometry (Å, °)

*D*—H⋯*A*	*D*—H	H⋯*A*	*D*⋯*A*	*D*—H⋯*A*
O11—H11*O*⋯O19	0.83 (2)	1.81 (2)	2.5766 (16)	153 (2)
O18—H18*O*⋯O19	0.83 (2)	1.89 (2)	2.6097 (16)	144 (2)
O11—H11*O*⋯O19^i^	0.83 (2)	2.40 (2)	2.8730 (16)	117 (2)
O18—H18*O*⋯O11^i^	0.83 (2)	2.35 (2)	2.9677 (17)	131 (2)
C3—H3⋯O20^ii^	0.95	2.25	3.1657 (18)	161
C13—H13⋯O1^iii^	0.95	2.46	3.348 (2)	156
C15—H15⋯O4^iv^	0.95	2.56	3.4358 (18)	153
C17—H17⋯O4^v^	0.95	2.43	3.228 (2)	141

Symmetry codes: (i) 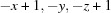; (ii) 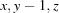; (iii) 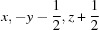; (iv) 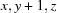; (v) 
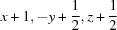
.

**Table 2:** Summary of short inter-atomic contacts (Å) in (I)

Contact	distance	symmetry operation
C11⋯C20	3.299 (2)	*x*, −1 + *y*, *z*
C15⋯C19	3.347 (2)	*x*, 1 + *y*, *z*
C4⋯O20	3.0273 (18)	*x*, *y*, *z*
C20⋯O4	3.1585 (18)	*x*, *y*, *z*
O18⋯H5	2.60	1 − *x*, − 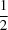 + *y*, 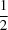 − *z*
C16⋯H16	2.89	1 − *x*, − 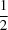 + *y*, 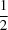 − *z*
H8⋯H8	2.27	-*x*, 2 − *y*, −*z*

**Table 3:** Percentage contribution of inter-atomic contacts to the Hirshfeld surface for (I)

Contact	percentage contribution		
	naphtho­quinone	anthra­quinone	(I)
H⋯H	20.5	21.4	20.6
O⋯H/H⋯O	29.2	28.4	31.3
C⋯H/H⋯C	15.2	25.2	20.2
C⋯C	9.7	7.1	9.3
C⋯O/O⋯C	3.9	11.9	5.4
Br⋯H/H⋯Br	10.0	4.1	6.5
Br⋯Br	4.6	0.0	2.4
Br⋯C/C⋯Br	5.2	0.0	2.8
Br⋯O/O⋯Br	1.1	0.1	0.7
O⋯O	0.5	1.8	0.8

**Table 4:** Summary of C=O⋯π contacts (Å, °) in (I) *Cg*1 and *Cg*2 are the centroids of the C11–C14/C24/C23 and C15–C18/C22/C21 rings, respectively.

*Y*	*X*	*Cg*	*X*⋯*Cg*	*Y*—*X*⋯*Cg*	*Y*⋯*Cg*	symmetry operation
C20	O20	*Cg*1	3.2667 (12)	85.61 (8)	3.3999 (16)	*x*, 1 + *y*, *z*
C19	O19	*Cg*2	3.3191 (12)	85.51 (8)	3.4551 (16)	*x*, −1 + *y*, *z*

**Table 5:** Experimental details

Crystal data	
Chemical formula	C_10_H_5_BrO_2_·C_14_H_8_O_4_
*M*_r_	477.25
Crystal system, space group	Monoclinic, *P*2_1_/*c*
Temperature (K)	100
*a*, *b*, *c* (Å)	17.55090 (12), 4.85939 (3), 22.83423 (16)
β (°)	106.7429 (7)
*V* (Å^3^)	1864.90 (2)
*Z*	4
Radiation type	Cu *K*α
μ (mm^−1^)	3.39
Crystal size (mm)	0.42 × 0.05 × 0.03

Data collection	
Diffractometer	Rigaku Saturn724+ (2x2 bin mode)
Absorption correction	Multi-scan (*CrysAlis PRO*; Rigaku Oxford Diffraction, 2015 ▸)
*T*_min_, *T*_max_	0.697, 1.000
No. of measured, independent and observed [*I* > 2σ(*I*)] reflections	27708, 3507, 3489
*R*_int_	0.021
(sin θ/λ)_max_ (Å^−1^)	0.610

Refinement	
*R*[*F* ^2^ > 2σ(*F* ^2^)], *wR*(*F* ^2^), *S*	0.025, 0.075, 1.02
No. of reflections	3507
No. of parameters	286
No. of restraints	2
H-atom treatment	H atoms treated by a mixture of independent and constrained refinement
Δρ_max_, Δρ_min_ (e Å^−3^)	0.39, −0.32

Computer programs: *CrysAlis PRO* (Rigaku Oxford Diffraction, 2015 ▸), *SHELXS* (Sheldrick, 2008 ▸), *SHELXL2014* (Sheldrick, 2015 ▸), *ORTEP-3 for Windows* (Farrugia, 2012 ▸), *DIAMOND* (Brandenburg, 2006 ▸) and *publCIF* (Westrip, 2010 ▸).

## supplementary crystallographic information

### Crystal data

**Table:**

C_10_H_5_BrO_2_·C_14_H_8_O_4_	*F*(000) = 960
*M**_r_* = 477.25	*D*_x_ = 1.700 Mg m^−^^3^
Monoclinic, *P*2_1_/*c*	Cu *K*α radiation, λ = 1.54184 Å
*a* = 17.55090 (12) Å	Cell parameters from 22842 reflections
*b* = 4.85939 (3) Å	θ = 2.6–69.9°
*c* = 22.83423 (16) Å	µ = 3.39 mm^−^^1^
β = 106.7429 (7)°	*T* = 100 K
*V* = 1864.90 (2) Å^3^	Plate, orange
*Z* = 4	0.42 × 0.05 × 0.03 mm

### Data collection

**Table:**

Rigaku Saturn724+ (2x2 bin mode) diffractometer	3507 independent reflections
Radiation source: fine-focus sealed X-ray tube, Enhance (Cu) X-ray Source	3489 reflections with *I* > 2σ(*I*)
Graphite monochromator	*R*_int_ = 0.021
ω scans	θ_max_ = 70.2°, θ_min_ = 2.6°
Absorption correction: multi-scan (CrysAlis PRO; Rigaku Oxford Diffraction, 2015)	*h* = −21→21
*T*_min_ = 0.697, *T*_max_ = 1.000	*k* = −5→4
27708 measured reflections	*l* = −27→27

### Refinement

**Table:**

Refinement on *F*^2^	2 restraints
Least-squares matrix: full	H atoms treated by a mixture of independent and constrained refinement
*R*[*F*^2^ > 2σ(*F*^2^)] = 0.025	*w* = 1/[σ^2^(*F*_o_^2^) + (0.0507*P*)^2^ + 1.0878*P*] where *P* = (*F*_o_^2^ + 2*F*_c_^2^)/3
*wR*(*F*^2^) = 0.075	(Δ/σ)_max_ < 0.001
*S* = 1.02	Δρ_max_ = 0.39 e Å^−^^3^
3507 reflections	Δρ_min_ = −0.32 e Å^−^^3^
286 parameters	

### Special details

**Table:**

Geometry. All esds (except the esd in the dihedral angle between two l.s. planes) are estimated using the full covariance matrix. The cell esds are taken into account individually in the estimation of esds in distances, angles and torsion angles; correlations between esds in cell parameters are only used when they are defined by crystal symmetry. An approximate (isotropic) treatment of cell esds is used for estimating esds involving l.s. planes.

### Fractional atomic coordinates and isotropic or equivalent isotropic displacement parameters (Å^2^)

**Table:**

	*x*	*y*	*z*	*U*_iso_*/*U*_eq_	
Br1	0.03352 (2)	0.06602 (3)	0.19884 (2)	0.02850 (9)	
O1	−0.00432 (7)	0.4511 (2)	0.08868 (6)	0.0283 (3)	
O4	0.30880 (7)	0.4406 (2)	0.20139 (5)	0.0240 (3)	
C1	0.06668 (9)	0.4618 (3)	0.11528 (7)	0.0212 (3)	
C2	0.10318 (9)	0.2887 (3)	0.17016 (7)	0.0212 (3)	
C3	0.18140 (9)	0.2861 (3)	0.19828 (7)	0.0220 (3)	
H3	0.2015	0.1702	0.2328	0.026*	
C4	0.23713 (9)	0.4592 (3)	0.17689 (7)	0.0193 (3)	
C4A	0.20374 (8)	0.6514 (3)	0.12522 (6)	0.0189 (3)	
C5	0.25340 (9)	0.8282 (3)	0.10564 (7)	0.0221 (3)	
H5	0.3089	0.8299	0.1259	0.027*	
C6	0.22192 (10)	1.0033 (4)	0.05623 (7)	0.0255 (3)	
H6	0.2558	1.1259	0.0431	0.031*	
C7	0.14060 (11)	0.9984 (4)	0.02611 (8)	0.0271 (3)	
H7	0.1192	1.1172	−0.0077	0.032*	
C8	0.09098 (9)	0.8207 (3)	0.04535 (7)	0.0248 (3)	
H8	0.0357	0.8171	0.0244	0.030*	
C8A	0.12158 (9)	0.6468 (3)	0.09523 (7)	0.0198 (3)	
O11	0.36741 (7)	−0.0561 (2)	0.48553 (5)	0.0244 (3)	
H11O	0.4113 (8)	0.004 (5)	0.4841 (10)	0.037*	
O18	0.58338 (6)	0.5207 (3)	0.42739 (5)	0.0245 (2)	
H18O	0.5660 (13)	0.396 (4)	0.4450 (9)	0.037*	
O19	0.47299 (6)	0.2395 (2)	0.45660 (5)	0.0217 (2)	
O20	0.24399 (6)	0.7932 (2)	0.29021 (5)	0.0261 (2)	
C11	0.31370 (9)	0.0927 (3)	0.44373 (7)	0.0199 (3)	
C12	0.23328 (10)	0.0292 (3)	0.43377 (7)	0.0229 (3)	
H12	0.2184	−0.1123	0.4570	0.027*	
C13	0.17525 (9)	0.1711 (4)	0.39025 (7)	0.0251 (3)	
H13	0.1208	0.1253	0.3837	0.030*	
C14	0.19585 (9)	0.3800 (4)	0.35607 (7)	0.0234 (3)	
H14	0.1556	0.4771	0.3265	0.028*	
C15	0.40221 (10)	0.9337 (3)	0.30130 (7)	0.0230 (3)	
H15	0.3619	1.0299	0.2716	0.028*	
C16	0.48208 (11)	0.9953 (4)	0.30923 (7)	0.0251 (3)	
H16	0.4960	1.1350	0.2851	0.030*	
C17	0.54127 (9)	0.8551 (3)	0.35188 (7)	0.0236 (3)	
H17	0.5955	0.8990	0.3568	0.028*	
C18	0.52207 (9)	0.6495 (3)	0.38783 (7)	0.0206 (3)	
C19	0.41987 (9)	0.3695 (3)	0.41798 (6)	0.0183 (3)	
C20	0.29639 (9)	0.6680 (3)	0.32790 (6)	0.0200 (3)	
C21	0.38191 (9)	0.7318 (3)	0.33688 (7)	0.0197 (3)	
C22	0.44137 (9)	0.5846 (3)	0.38098 (7)	0.0179 (3)	
C23	0.33587 (8)	0.3038 (3)	0.40931 (6)	0.0180 (3)	
C24	0.27558 (9)	0.4463 (3)	0.36537 (7)	0.0193 (3)	

### Atomic displacement parameters (Å^2^)

**Table:**

	*U*^11^	*U*^22^	*U*^33^	*U*^12^	*U*^13^	*U*^23^
Br1	0.02564 (12)	0.02819 (13)	0.03685 (13)	−0.00434 (6)	0.01723 (9)	−0.00040 (6)
O1	0.0168 (5)	0.0350 (7)	0.0321 (6)	0.0009 (4)	0.0054 (5)	−0.0024 (5)
O4	0.0182 (5)	0.0298 (7)	0.0237 (5)	0.0035 (4)	0.0055 (4)	0.0024 (4)
C1	0.0180 (7)	0.0225 (8)	0.0239 (7)	0.0026 (6)	0.0072 (6)	−0.0046 (6)
C2	0.0216 (7)	0.0202 (7)	0.0250 (7)	−0.0003 (6)	0.0118 (6)	−0.0018 (6)
C3	0.0239 (7)	0.0212 (7)	0.0220 (7)	0.0032 (6)	0.0085 (6)	0.0019 (6)
C4	0.0194 (7)	0.0199 (8)	0.0194 (7)	0.0020 (6)	0.0070 (6)	−0.0017 (5)
C4A	0.0179 (7)	0.0195 (7)	0.0201 (7)	0.0035 (6)	0.0066 (5)	−0.0010 (6)
C5	0.0211 (7)	0.0226 (8)	0.0242 (7)	0.0031 (6)	0.0091 (6)	−0.0004 (6)
C6	0.0303 (9)	0.0227 (7)	0.0277 (8)	0.0036 (7)	0.0151 (7)	0.0023 (7)
C7	0.0327 (9)	0.0261 (8)	0.0242 (8)	0.0094 (7)	0.0111 (7)	0.0046 (7)
C8	0.0233 (7)	0.0269 (8)	0.0229 (7)	0.0067 (6)	0.0049 (6)	0.0016 (6)
C8A	0.0180 (7)	0.0205 (7)	0.0212 (7)	0.0038 (6)	0.0064 (5)	−0.0019 (6)
O11	0.0228 (6)	0.0244 (6)	0.0265 (6)	−0.0019 (4)	0.0077 (5)	0.0069 (4)
O18	0.0180 (5)	0.0250 (6)	0.0315 (6)	−0.0010 (5)	0.0086 (4)	0.0047 (5)
O19	0.0182 (5)	0.0232 (5)	0.0233 (5)	0.0006 (4)	0.0052 (4)	0.0045 (4)
O20	0.0241 (5)	0.0279 (6)	0.0257 (5)	0.0058 (5)	0.0061 (4)	0.0049 (5)
C11	0.0211 (8)	0.0196 (7)	0.0193 (7)	−0.0003 (6)	0.0064 (6)	−0.0039 (5)
C12	0.0241 (8)	0.0239 (8)	0.0241 (7)	−0.0051 (6)	0.0125 (6)	−0.0023 (6)
C13	0.0186 (7)	0.0298 (9)	0.0294 (8)	−0.0038 (6)	0.0109 (6)	−0.0055 (7)
C14	0.0190 (7)	0.0272 (8)	0.0235 (7)	0.0019 (6)	0.0053 (6)	−0.0030 (6)
C15	0.0293 (8)	0.0195 (8)	0.0220 (7)	0.0024 (6)	0.0100 (6)	0.0006 (5)
C16	0.0352 (8)	0.0202 (7)	0.0253 (8)	−0.0024 (7)	0.0172 (7)	0.0009 (7)
C17	0.0233 (7)	0.0238 (8)	0.0280 (8)	−0.0037 (6)	0.0144 (6)	−0.0035 (7)
C18	0.0210 (7)	0.0195 (7)	0.0228 (7)	−0.0002 (6)	0.0088 (6)	−0.0039 (6)
C19	0.0196 (7)	0.0176 (7)	0.0185 (7)	0.0002 (6)	0.0066 (5)	−0.0032 (6)
C20	0.0226 (7)	0.0196 (7)	0.0184 (7)	0.0021 (6)	0.0068 (6)	−0.0016 (6)
C21	0.0225 (7)	0.0184 (7)	0.0197 (7)	0.0006 (6)	0.0085 (5)	−0.0019 (6)
C22	0.0194 (7)	0.0172 (7)	0.0190 (7)	−0.0005 (5)	0.0083 (6)	−0.0022 (5)
C23	0.0185 (7)	0.0179 (7)	0.0187 (6)	−0.0006 (6)	0.0072 (5)	−0.0020 (5)
C24	0.0196 (7)	0.0201 (8)	0.0195 (7)	0.0004 (5)	0.0074 (6)	−0.0026 (5)

### Geometric parameters (Å, º)

**Table:**

Br1—C2	1.8857 (15)	O20—C20	1.2248 (18)
O1—C1	1.220 (2)	C11—C12	1.398 (2)
O4—C4	1.2239 (19)	C11—C23	1.413 (2)
C1—C8A	1.483 (2)	C12—C13	1.385 (2)
C1—C2	1.492 (2)	C12—H12	0.9500
C2—C3	1.338 (2)	C13—C14	1.390 (2)
C3—C4	1.476 (2)	C13—H13	0.9500
C3—H3	0.9500	C14—C24	1.391 (2)
C4—C4A	1.486 (2)	C14—H14	0.9500
C4A—C5	1.386 (2)	C15—C21	1.384 (2)
C4A—C8A	1.407 (2)	C15—C16	1.393 (2)
C5—C6	1.394 (2)	C15—H15	0.9500
C5—H5	0.9500	C16—C17	1.382 (2)
C6—C7	1.395 (2)	C16—H16	0.9500
C6—H6	0.9500	C17—C18	1.395 (2)
C7—C8	1.385 (3)	C17—H17	0.9500
C7—H7	0.9500	C18—C22	1.415 (2)
C8—C8A	1.395 (2)	C19—C22	1.460 (2)
C8—H8	0.9500	C19—C23	1.465 (2)
O11—C11	1.3433 (19)	C20—C24	1.485 (2)
O11—H11O	0.833 (10)	C20—C21	1.488 (2)
O18—C18	1.3436 (19)	C21—C22	1.417 (2)
O18—H18O	0.831 (10)	C23—C24	1.412 (2)
O19—C19	1.2541 (18)		
			
O1—C1—C8A	122.25 (15)	C11—C12—H12	119.8
O1—C1—C2	121.59 (15)	C12—C13—C14	120.66 (14)
C8A—C1—C2	116.16 (13)	C12—C13—H13	119.7
C3—C2—C1	122.79 (14)	C14—C13—H13	119.7
C3—C2—Br1	120.42 (12)	C24—C14—C13	119.74 (14)
C1—C2—Br1	116.79 (11)	C24—C14—H14	120.1
C2—C3—C4	121.47 (14)	C13—C14—H14	120.1
C2—C3—H3	119.3	C21—C15—C16	119.69 (15)
C4—C3—H3	119.3	C21—C15—H15	120.2
O4—C4—C3	119.89 (14)	C16—C15—H15	120.2
O4—C4—C4A	121.84 (14)	C17—C16—C15	120.65 (15)
C3—C4—C4A	118.26 (13)	C17—C16—H16	119.7
C5—C4A—C8A	120.29 (14)	C15—C16—H16	119.7
C5—C4A—C4	120.30 (13)	C16—C17—C18	120.53 (15)
C8A—C4A—C4	119.38 (14)	C16—C17—H17	119.7
C4A—C5—C6	119.99 (14)	C18—C17—H17	119.7
C4A—C5—H5	120.0	O18—C18—C17	116.53 (14)
C6—C5—H5	120.0	O18—C18—C22	123.57 (14)
C7—C6—C5	119.96 (16)	C17—C18—C22	119.90 (14)
C7—C6—H6	120.0	O19—C19—C22	120.26 (13)
C5—C6—H6	120.0	O19—C19—C23	120.02 (14)
C8—C7—C6	120.14 (15)	C22—C19—C23	119.71 (13)
C8—C7—H7	119.9	O20—C20—C24	120.36 (14)
C6—C7—H7	119.9	O20—C20—C21	121.12 (14)
C7—C8—C8A	120.47 (15)	C24—C20—C21	118.52 (13)
C7—C8—H8	119.8	C15—C21—C22	120.88 (14)
C8A—C8—H8	119.8	C15—C21—C20	119.13 (14)
C8—C8A—C4A	119.15 (15)	C22—C21—C20	119.99 (14)
C8—C8A—C1	119.16 (14)	C18—C22—C21	118.35 (14)
C4A—C8A—C1	121.69 (14)	C18—C22—C19	120.85 (14)
C11—O11—H11O	104.5 (16)	C21—C22—C19	120.80 (14)
C18—O18—H18O	109.2 (16)	C24—C23—C11	118.74 (13)
O11—C11—C12	117.75 (14)	C24—C23—C19	120.53 (13)
O11—C11—C23	122.46 (14)	C11—C23—C19	120.71 (13)
C12—C11—C23	119.78 (14)	C14—C24—C23	120.66 (14)
C13—C12—C11	120.41 (15)	C14—C24—C20	118.90 (14)
C13—C12—H12	119.8	C23—C24—C20	120.44 (13)
			
O1—C1—C2—C3	176.47 (15)	C16—C15—C21—C20	−179.96 (14)
C8A—C1—C2—C3	−3.6 (2)	O20—C20—C21—C15	−0.9 (2)
O1—C1—C2—Br1	−3.0 (2)	C24—C20—C21—C15	178.56 (13)
C8A—C1—C2—Br1	176.89 (11)	O20—C20—C21—C22	179.75 (14)
C1—C2—C3—C4	0.7 (2)	C24—C20—C21—C22	−0.8 (2)
Br1—C2—C3—C4	−179.76 (11)	O18—C18—C22—C21	−178.89 (14)
C2—C3—C4—O4	−175.82 (15)	C17—C18—C22—C21	0.4 (2)
C2—C3—C4—C4A	3.7 (2)	O18—C18—C22—C19	0.5 (2)
O4—C4—C4A—C5	−3.8 (2)	C17—C18—C22—C19	179.80 (14)
C3—C4—C4A—C5	176.67 (14)	C15—C21—C22—C18	0.1 (2)
O4—C4—C4A—C8A	174.35 (14)	C20—C21—C22—C18	179.47 (13)
C3—C4—C4A—C8A	−5.2 (2)	C15—C21—C22—C19	−179.30 (14)
C8A—C4A—C5—C6	0.3 (2)	C20—C21—C22—C19	0.1 (2)
C4—C4A—C5—C6	178.50 (14)	O19—C19—C22—C18	0.2 (2)
C4A—C5—C6—C7	−0.7 (2)	C23—C19—C22—C18	−179.10 (13)
C5—C6—C7—C8	0.3 (3)	O19—C19—C22—C21	179.56 (13)
C6—C7—C8—C8A	0.5 (3)	C23—C19—C22—C21	0.3 (2)
C7—C8—C8A—C4A	−0.9 (2)	O11—C11—C23—C24	178.68 (13)
C7—C8—C8A—C1	179.08 (15)	C12—C11—C23—C24	0.0 (2)
C5—C4A—C8A—C8	0.5 (2)	O11—C11—C23—C19	0.1 (2)
C4—C4A—C8A—C8	−177.71 (14)	C12—C11—C23—C19	−178.57 (14)
C5—C4A—C8A—C1	−179.51 (14)	O19—C19—C23—C24	−179.12 (13)
C4—C4A—C8A—C1	2.3 (2)	C22—C19—C23—C24	0.2 (2)
O1—C1—C8A—C8	1.9 (2)	O19—C19—C23—C11	−0.6 (2)
C2—C1—C8A—C8	−178.02 (14)	C22—C19—C23—C11	178.72 (13)
O1—C1—C8A—C4A	−178.11 (15)	C13—C14—C24—C23	−0.1 (2)
C2—C1—C8A—C4A	2.0 (2)	C13—C14—C24—C20	179.34 (14)
O11—C11—C12—C13	−178.50 (14)	C11—C23—C24—C14	−0.1 (2)
C23—C11—C12—C13	0.2 (2)	C19—C23—C24—C14	178.52 (14)
C11—C12—C13—C14	−0.4 (2)	C11—C23—C24—C20	−179.52 (13)
C12—C13—C14—C24	0.4 (2)	C19—C23—C24—C20	−0.9 (2)
C21—C15—C16—C17	0.6 (2)	O20—C20—C24—C14	1.2 (2)
C15—C16—C17—C18	−0.1 (2)	C21—C20—C24—C14	−178.20 (13)
C16—C17—C18—O18	178.93 (14)	O20—C20—C24—C23	−179.31 (14)
C16—C17—C18—C22	−0.4 (2)	C21—C20—C24—C23	1.3 (2)
C16—C15—C21—C22	−0.6 (2)		

### Hydrogen-bond geometry (Å, º)

**Table:**

*D*—H···*A*	*D*—H	H···*A*	*D*···*A*	*D*—H···*A*
O11—H11*O*···O19	0.83 (2)	1.81 (2)	2.5766 (16)	153 (2)
O18—H18*O*···O19	0.83 (2)	1.89 (2)	2.6097 (16)	144 (2)
O11—H11*O*···O19^i^	0.83 (2)	2.40 (2)	2.8730 (16)	117 (2)
O18—H18*O*···O11^i^	0.83 (2)	2.35 (2)	2.9677 (17)	131 (2)
C3—H3···O20^ii^	0.95	2.25	3.1657 (18)	161
C13—H13···O1^iii^	0.95	2.46	3.348 (2)	156
C15—H15···O4^iv^	0.95	2.56	3.4358 (18)	153
C17—H17···O4^v^	0.95	2.43	3.228 (2)	141

Symmetry codes: (i) −*x*+1, −*y*, −*z*+1; (ii) *x*, *y*−1, *z*; (iii) *x*, −*y*−1/2, *z*+1/2; (iv) *x*, *y*+1, *z*; (v) *x*+1, −*y*+1/2, *z*+1/2.
